# Reasons for failure of prevention of mother-to-child HIV transmission in a rural South African district hospital

**DOI:** 10.4102/sajhivmed.v16i1.365

**Published:** 2015-04-28

**Authors:** Clifford Kendall, Lore Claessens, Jienchi Dorward, Gloria Mfeka, Kelly Gate

**Affiliations:** 1 Bethesda Hospital, Umkhanyakude District, KwaZulu-Natal, South Africa; 2 20,000 Plus Partnership, University of KwaZulu-Natal, South Africa; 3 Department of Family Medicine, University of KwaZulu-Natal, South Africa

## Abstract

Further reduction of mother-to-child transmission (MTCT) of HIV requires improved understanding of the reasons for MTCT. We reviewed maternal and infant case notes for HIV-positive infants diagnosed by polymerase chain reaction at Bethesda Hospital. Nineteen cases were analysed. Median gestation at first antenatal consultation (ANC) was 22.5 (interquartile range [IQR] 19.25–24). Eleven (57.9%) mothers were HIV positive at first ANC, whilst eight tested negative and later positive (2 antepartum, 6 postpartum). Median maternal CD4 was 408 cells/μL (IQR 318–531). Six (31.6%) received no antenatal antiretroviral therapy (ART) because they were diagnosed as HIV positive postpartum; 9 (47.3%) received antenatal ART and 3 (15.8%) were never initiated on ART. At 6 weeks postpartum, 5 infants (26.3%) were not on prophylactic nevirapine (NVP) because their mothers had not yet been diagnosed. Maternal seroconversion in pregnancy and breastfeeding, and possibly false-negative HIV tests, were important reasons for prevention of mother-to-child transmission (PMTCT) failure.

## Introduction

In 2010, the South African Department of Health (DoH) prevention of mother-to-child transmission (PMTCT) guidelines recommended World Health Organization Option A (prophylactic zidovudine [AZT] for women with a CD4+ count > 350 cells/μL and combination antiretroviral therapy [cART] for all pregnant women with CD4 < 350 cells/μL, with subsequent infant nevirapine [NVP] for a minimum of 6 weeks).^1^ Option B (cART for all pregnant and breastfeeding women irrespective of CD4 count and postnatal infant NVP prophylaxis) was introduced in April 2013.^2^ Using these guidelines, mother-to-child transmission (MTCT) in KwaZulu-Natal, South Africa, dropped from 20.8% at 6 weeks postpartum in 2005 to 2.1% in 2011,^3,4^ with a national target of less than 2% by 2016.^5^ Further reduction will require a better understanding of the reasons for PMTCT failure in local facilities. Seroconversion in pregnancy or breastfeeding, HIV diagnosis in pregnancy compared with diagnosis prior to conception, and health system-related factors have all been found to play a role in PMTCT failure.^6,7,8^

Bethesda is a rural district hospital serving approximately 115 000 people in Umkhanyakude District, KwaZulu-Natal Province, with an HIV prevalence of 41.1% amongst pregnant women in 2011.^9^ HIV polymerase chain reaction (PCR) positivity at 6 weeks postpartum in 2013 was 2.3% for Bethesda Hospital (personal comm., Facility Information Officer, n.d.) and its eight peripheral primary healthcare clinics. Our aim was to identify reasons for these PMTCT failures.

## Methods

We retrospectively reviewed maternal and infant case notes for HIV-positive infants identified by HIV PCR between February and September 2013 at Bethesda Hospital and its clinics.

### Ethics approval

Ethics approval for the study was granted by the University of KwaZulu-Natal Biomedical Research Ethics Committee and the KwaZulu-Natal Health Research Committee.

## Results

A total of 25 cases of MTCT were identified in the study period. Data were available for analysis in 19 cases (Table 1). Notes were often incomplete, meaning data were not available for all 19 cases for some variables. Median maternal age was 22 years (interquartile range [IQR] 20.5–28). Median gestation at first antenatal consultation (ANC) was 22.5 weeks (IQR 19.25–24) and 9 (47.3%) women were known to have had their first ANC after 20 weeks’ gestation. Five (26.3%) women were known to be HIV positive preconception. A further 6 (31.6%) tested HIV positive at first ANC. Eight (42.1%) tested HIV negative at first ANC, but two of these subsequently tested positive antenatally (1 and 3 weeks before delivery respectively). The remaining 6 (31.6%) women tested HIV positive postpartum. Median maternal CD4 at baseline was 408 cells/μL (IQR 318–531). Of the 13 who were known to be HIV positive before delivery, 1/13 (7.7%) had unknown antenatal antiretroviral therapy (ART) status, 3/13 (23.1%) were never initiated on ART before delivery, 3/13 (23.1%) were already on cART pre-conception, and 6/13 (46.2%) were initiated on ART antenatally (cART = 4, AZT monotherapy = 2) at a median of 28 weeks’ gestation (IQR 26–30) and 0 days (IQR 0–16) after being diagnosed as requiring PMTCT. One of these patients had a documented history of poor adherence/defaulting. The six patients diagnosed postpartum did not have information on maternal ART initiation available. Of the three patients on cART pre-conception, 2 had viral loads taken antenatally and both were greater than 400 copies/mL. Five women had caesarean sections.

**FIGURE 1:**
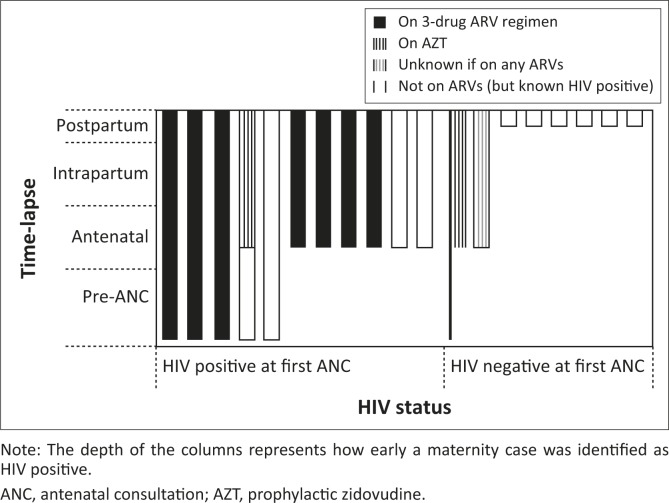
Period of maternal HIV diagnosis and initiation

**TABLE 1:** Maternal and infant characteristics.

Characteristics	Sub-characteristics	*n*	%	Median	IQR
Variable	Maternal age (years)	19	-	22	20.5–28
	Gestation at first ANC (weeks)	14 (5 unknown)	-	22.5	19.3–24
	Maternal CD4 (cells/μL)	14 (5 unknown)	-	408	318–531
	Weeks from infant diagnosis to ART initiation	13 (6 unknown)	-	5	3–11
Maternal HIV status at first ANC	Positive	11	57.9	-	-
	Negative	8	42.1	-	-
Period of maternal HIV diagnosis	Pre-conception	5	26.3	-	-
	Antenatal	8	42.1	-	-
	Postpartum	6	31.6	-	-
Antenatal ART regimen	None because diagnosed postnatally	6	31.6	-	-
	cART	7	36.8	-	-
	Prophylactic AZT	2	10.5	-	-
	No ART	3	15.8	-	-
	Unknown	1	5.3	-	-
Delivery	Normal vaginal delivery	12	63.2	-	-
	Caesarean section	5	26.3	-	-
	Unknown	2	10.5	-	-
	Gestation at delivery (weeks)	14 (5 unknown)	-	38	36–38
Infant NVP prophylaxis at 6 weeks postpartum?	Yes	8	42.1	-	-
	No†	6†	31.6	-	-
	Unknown	5	26.3	-	-
Infant feeding method at 6 weeks postpartum?	Exclusive breastfeeding	8	42.1	-	-
	Exclusive formula feeding	3	15.8	-	-
	Mixed feeding	2	10.5	-	-
	Unknown	6	31.6	-	-

*N* = 19.

*n* = number of cases per category; ANC, antenatal consultation; ART, antenatal antiretroviral therapy; cART, combination antiretroviral therapy; AZT, prophylactic zidovudine; NVP, nevirapine; IQR, interquartile range.

†, Five infants were not on NVP at their 6-week postnatal follow-up visit because their mothers had not yet tested HIV positive.

Regarding infants, 5 (26.3%) were not on NVP at their 6-week postnatal follow-up visit because their mothers had not yet tested HIV positive. Of the remaining 14 subjects, only 8/14 (57.1%) infants were documented to be on NVP prophylaxis, with 6/14 (42.9%) having no record of NVP administered. Two (10.5%) infants were documented as receiving mixed feeding at 6 weeks. One (5.3%) infant died before cART initiation, and 13 (68.4%) were known to have been initiated on cART at a median 5 (IQR 3–11) weeks after diagnosis.

## Discussion

Maternal and infant ART have consistently been shown to be highly efficacious for PMTCT; consequently, omitting or delaying ART exposes infants to unnecessary HIV transmission risks.^10^ In the present study, we repeat findings elsewhere^6,8^ that initially testing HIV negative and subsequently positive in pregnancy or breastfeeding (leading to delays in ART initiation) is a major cause of PMTCT failure, occurring in 8 (42.1%) of the PMTCT failure cases in our study. We could not determine whether this was because of an initial false-negative test, or maternal seroconversion in the majority of cases. Current PMTCT guidelines advocate a confirmatory second HIV rapid test if the initial test is positive. However, there is no confirmatory test if the first test is negative.^2^ A study of 967 adults presenting for HIV testing in a clinic in Durban found that 2.1% of patients with a negative rapid HIV test had either acute HIV infection (which was missed by the rapid test because of falling within the ‘window period’) or chronic HIV infection (i.e. a false-negative rapid test).^11^ In the context of further reducing MTCT to below 2%, potentially misdiagnosing 2% of mothers living with HIV is significant. Strategies to increase detection of all positive cases could include a second confirmatory rapid test as routine in all pregnant women (with an HIV ELISA confirmatory test for all discordant results). Furthermore, regular 3-monthly maternal HIV testing throughout the duration of pregnancy and breastfeeding, in accordance with DoH Guidelines,^2^ or even reducing the repeat testing interval to 1- or 2–monthly, will be crucial to ensure that maternal seroconversion is detected as soon as possible.

Not documenting infant NVP prophylaxis when it is known to be indicated occurred in 6 (42.9%) cases in our study. However, this is probably higher than the true value as, from clinical experience, we note that infant NVP is often administered but poorly documented. Late booking after 20 weeks’ gestation (also leading to late initiation of maternal ART) occurred in 9 (47%) of cases, although 75% of cases had booked before 24 weeks which should allow time for viral suppression by the time of delivery, assuming ART is initiated promptly.^10,12^ Omitting maternal ART when it is known to be indicated occurred in 3 (15.8%) cases (we were unable to ascertain why), and virological failure despite maternal cART occurred in 2 (10.5%) cases. Both these cases had repeated viral loads > 400 copies/mL more than 1 month apart but were not switched to second-line cART. The third case had no viral load sampling during pregnancy.

Weaknesses of our study include lack of a control group, small sample size and incomplete or unavailable case notes. Data were too incomplete for analysis of several important variables (e.g. duration and means of rupture of membranes, elective versus emergency caesarean section, instrumental delivery, postpartum maternal ART adherence). Only descriptive analysis was possible and our results must be interpreted with caution.

## Conclusion

PMTCT remains a focus programme in the South African healthcare sector. Better understanding of the reasons for MTCT can assist further reduction of MTCT rates to the target of less than 2%. Several causes for the failure of PMTCT in our sub-district have been identified. These correspond with reasons for MTCT from previous studies and available literature. Late first ANC, delayed or omission of maternal cART initiation, and poor management of women on cART contributed, amongst other factors, to our MTCT cases. Maternal seroconversion or an initial false-negative HIV test occurs frequently in PMTCT failures in our clinics, with subsequent late maternal ART initiation. This fact highlights the importance of preventing and promptly detecting maternal HIV infection in pregnancy and breastfeeding if MTCT is to be further reduced. Further research is needed to characterise the frequency of false-negative HIV testing in operational PMTCT programmes, and to identify cost-effective testing strategies to ensure early detection of acute maternal HIV infection in pregnancy and breastfeeding.
